# Clinical application of endobronchial ultrasonography-guided transbronchial needle aspiration biopsy—a single center, large sample, real-world study

**DOI:** 10.1186/s12890-023-02568-4

**Published:** 2023-09-09

**Authors:** Chun-li Tang, Zheng Zhu, Chang-hao Zhong, Zi-qing Zhou, Hui-qi Zhou, Rong-mei Geng, Xiao-bo Chen, Yu Chen, Shi-yue Li

**Affiliations:** 1 Department of Respiratory and Critical Care Medicine, National Center for Respiratory Medicine, State Key Lab of Respiratory Disease, The first affiliated hospital of Guangzhou Medical University, 151 Yanjiang Road, Guangzhou, 510120 China; 2 Department of Allergy and Clinical Immunology, National Center for Respiratory Medicine, State Key Lab of Respiratory Disease, The first affiliated hospital of Guangzhou Medical University, Guangzhou, China

**Keywords:** EBUS-TBNA, Diagnostic value, Biopsy, Lymph node

## Abstract

**Background:**

Endobronchial ultrasonography-guided transbronchial needle aspiration biopsy (EBUS-TBNA) has been used for more than 10 years in China. Its clinical application and diagnostic value in different diseases with large sample was lack of report.

**Methods:**

A retrospective analysis was performed about the application and diagnostic value of EBUS-TBNA in different disease of patients in Respiratory Intervention Center of Guangzhou Institute of Respiratory Health from January 2012 to July 2020.

**Results:**

A total 5758 patients were included with 182 patients excluded for lack of information. Finally, data of 5576 patients (3798 males and 1778 females) were analyzed. For anesthetize, most patients were undergoing general anesthesia of intravenous with spontaneous breathing (69.4%), followed by general anesthesia of intravenous and inhalation with tracheal intubation and mechanical ventilation (17.9%) and conscious sedation and analgesia (12.8%). Lymph nodes were the main sites of biopsy obtained (76.4%). Tumors accounted for the highest proportion of disease (66.4%), followed by infection diseases (9.9%), sarcoidosis (3.9%), lymphoma (1.1%), and others (18.7%). The sensitivity of EBUS-TBNA for diagnosis of tumor was 89.7%, and 40.8% for infection diseases. There were significant differences in the puncture site and proportions of diseases between male and females (both p < 0.05). Higher diagnostic value was found in male patients (p < 0.05).

**Conclusion:**

EBUS-TBNA has good diagnostic value for different mediastinal and central pulmonary space-occupying lesions diseases, with highest sensitivity for tumors. Higher diagnostic value was found in male patients.

## Introduction

Endobronchial ultrasound-guided transbronchial needle aspiration (EBUS-TBNA) is a useful technology developed in the past 20 years, has been recommended as a safe and important tool for preoperative evaluation of lung cancer by American National Cancer Network and the American College of Chest Physicians since 2007 [1, 2], and has been clinically applied in China since 2008. The main indications of EBUS-TBNA include: diagnosis of intrapulmonary tumors; lymph node (LN) staging in patients with lung cancer; diagnosis of unexplained hilar and/or mediastinal LN enlargement; and diagnosis of mediastinal tumors. The application of EBUS-TBNA has greatly improved the sensitivity and specificity of transtracheal and bronchial needle biopsy [3, 4]. In China, it was recommended in Adult Diagnostic Flexible Bronchoscopy Application Guidelines (2019 Edition): (1) For extra-bronchial lesions, TBNA or EBUS-TBNA to improve the positive rate; (2) For suspected of sarcoidosis, mucosal biopsy, TBLB combined with CD4+/CD8 + ratio detection in bronchoalveolar lavage fluid, if the mediastinal lymph nodes are enlarged, combined with TBNA or EBUS-TBNA to increase the positive rate of diagnosis [5–10].

At present, there has not a guideline or consensus on the use of EBUS-TBNA, and each unit is carried out after the technical support of the manufacturer and the training of other training unit/center. Its clinical application lacks large sample reports, especially for a certain type of or a certain disease. It reported that the sensitivity of EBUS-TBNA in the diagnosis of central lung cancer or mediastinal space-occupying lesions is about 60-90% [4, 11]. Factors that affect the positive rate of EBUS-TBNA include the patient’s condition (lesion location, size, tolerance for anesthesia), experience of operator, pathological examination technique, and equipment. There are few reports on the safety and complications of EBUS-TBNA, which showed that EBUS-TBNA is generally safe, mainly complications including bleeding at the puncture site and infection [12].

As one of the earliest units that carry out EBUS-TBNA in China, Respiratory Intervention Center of Guangzhou Institute of Respiratory Health has accumulated large sample of patients that received EBUS-TBNA in the past ten years. In order to better understand the clinical application of EBUS-TBNA and better guide clinical practice, in this retrospective study, we analyzed the application and diagnostic value of EBUS-TBNA in different diseases.

## Methods

This is a retrospective study. Statistical analysis was performed on the data of patients who underwent EBUS-TBNA in the Respiratory Intervention Center of Guangzhou Institute of Respiratory Health from January 2012 to July 2020. All patients signed informed consent for EBUS-TBNA, and without related contraindications. This project was approved by the Ethics Committee of the First Affiliated Hospital of Guangzhou Medical University.

### General information

A total of 5758 patients completed EBUS-TBNA were included, and 182 were excluded for lack of pathological results or clinical information. Finally, data of 5576 patients were included and analyzed, including 3798 male patients and 1778 female patients, with an average age of 58.9 ± 15.3 years and 54.8 ± 13.9 years, respectively (Table 1).

**Table 1:** Characteristics of the patients and clinical information

	Total N(%)	Male N(%)	Female N(%)	P
Gander		3798	1778	< 0.01
Age, yrs.	57.6 ± 14.9	58.9 ± 15.3	54.8 ± 13.9	< 0.01
Clinical diagnosis				
Tumors	3705(66.4)	2675(70.4)	1030(57.9)	< 0.01
Infectious	554(9.9)	338(8.9)	216(12.1)	< 0.01
Sarcoidosis	215(3.9)	99(2.6)	116(6.5)	0.25
Lymphoma	61(1.1)	40(1.1)	21(1.2)	0.02
Unclear	1041(18.7)	646(17.1)	395(22.1)	< 0.01
Pathology results				
Tumor	3481(62.4)	2519(66.3)	962(54.1)	< 0.01
Infectious	234(4.2)	145(3.8)	89(5.0)	< 0.01
Sarcoidosis	174(3.1)	85(2.2)	89(5.0)	0.76
Lymphoma	39(0.7)	22(0.6)	17(1.0)	0.42
Unclarified	1648(29.5)	1027(27.0)	621(34.9)	< 0.01
Site of biopsy				
Lymph nodes	4263(76.5)	2937(77.3)	1326(74.6)	< 0.01
Intrapulmonary lesions	944(16.9)	619(16.3)	325(18.3)	< 0.01
Lymph-node and intrapulmonary lesions	193(3.5)	124(3.3)	69(3.9)	< 0.01
Mediastinal-mass and other site	176(3.2)	118(3.1)	58(3.2)	< 0.01

### EBUS-TBNA performance and result judgment

In the respiratory endoscopy center, patients signed the informed consent, and then underwent analgesia for the performance of EBUS-TBNA. CP-EBUS was only used with Endoscopic system: Olympus BF-UC260F-OL8, BF-UC260FW (Olympus Corporation, Japan), puncture needle, Olympus (21G, NA-201SX-4022, NA-201SX-4021)(Olympus Corporation, Japan). EBUS-TBNA were performed by experienced doctors, mediastinal and hilar lymph nodes, as well as centrally located parenchymal lesions and visible with endobronchial ultrasound were punctured by needles for 3–5 times, the sample obtained using EBUS -TBNA needle were tissue or cells by aspiration, which were fixed with formalin and sent to the pathology department for histopathological examinations.

The final diagnosis final diagnosis were confirmed by surgical biopsy and or by long-term follow-up and evaluation of treatment effects. In this study, all of the the patients were followed-up for 1 year.

### Anesthesia

Anesthesia were performed in the operating room by an anesthesiologist. General anesthesia of intravenous with spontaneous breathing using propofol, midazolam and sufentanil, while shallow sedation and analgesia with conscious spontaneous breathing using midazolam and sufentanil.

### Statistical analysis

Statistical analysis was performed by using SPSS 18.0 (Chicago USA). Normal distribution data were expressed as mean ± standard deviation (M ± SD), or M(IQ). t-test was used for comparison of two independent samples; enumeration data was expressed as rate N (%), and χ2 test was used. A p value < 0.05 means statistically significant difference.

## Results

### Characteristics of the patients

**Fig. 1:**
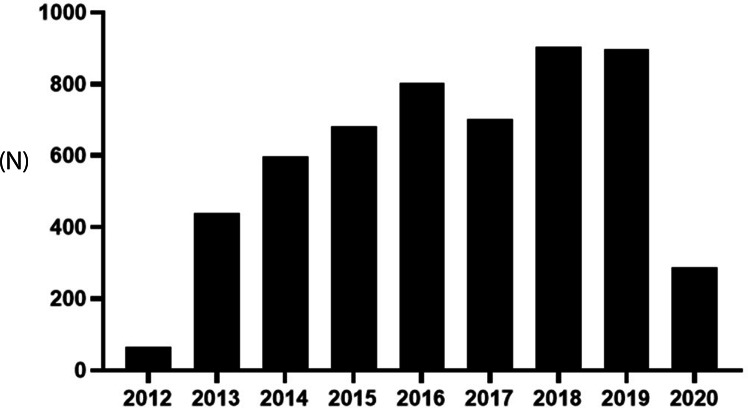
Number of EBUS-TBNA finished with year

**Fig. 2:**
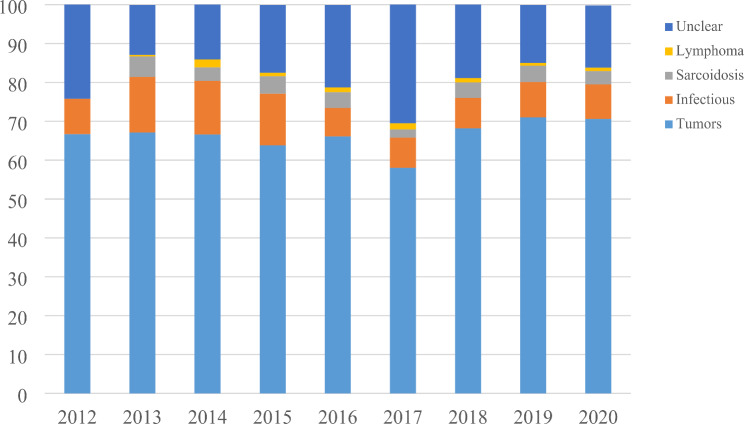
Distribution of diseases in different years

A total of 5576 patients finally included and analyzed in this study, the number of patients that completed EBUS-TBNA increased over the years (Fig. 1), lung tumors accounted for 66.4% (3705 cases), followed by pulmonary infectious diseases 9.9% (554 cases), Sarcoidosis was 3.9% (215 cases), lymphoma was 1.1% (61 cases), and unclear was about 18.7% (1041 cases) (Table 1). There was not significant different of the distribution or kinds of diseases in different years (Fig. 2). There were more males than females, and among male patients, tumor accounted for the highest proportion with 70.4% (2675 cases), followed by infectious diseases 8.9% (338 cases); while in female patients, tumor accounted for 57.9% (1030 cases), followed by infectious diseases12.1% (216 cases) (Table 1).

### Puncture site and results of pathology

The main puncture sites of biopsy were lymph nodes of 4263 patients (76.5%), intrapulmonary lesions of 944 patients (16.9%), both lymph node and intrapulmonary lesions of 193 patients (3.5%), and mediastinal mass and other sites of 176 cases (3.2%) (Table 1). However, up to 93% of the biopsy were taken from lymph-nodes in sarcoidosis patients; The highest proportion of biopsy obtained in intrapulmonary lesions (18.1%) was found in infection disease of patients (Table 2).

**Table 2:** distribution of biopsy sites of patients with different diseases

Site/Disease	Tumor N(%)	Infectious N(%)	Sarcoidosis N(%)	Lymphoma N(%)	Others N(%)	p
Lymph-node	2852(77.0)	431(77.8)	200(93.0)	44(72.1)	736(70.7)	< 0.01
Intrapulmonary lesions	599(16.2)	100(18.1)	7(0.7)	10(16.4)	228(21.9)	< 0.01
Lymph-node and intrapulmonary lesions	146(3.9)	13(2.3)	2(0.9)	1(1.6)	31(3.0)	< 0.01
Mediastinal-mass and other site	108(2.9)	10(1.8)	6(2.8)	6(9.8)	46(4.4)	< 0.01
Total	3705	554	215	61	1041	

The pathological results were neoplastic in 3481 cases (62.4%), infectious in 234 cases (4.2%), pulmonary sarcoidosis in 174 cases (3.1%), lymphoma in 39 cases (0.7%), and could not be clarified 1648 cases (29.5%). For male patients, 2519 cases (66.3%) were tumor diseases and 145 cases (3.8%) were infectious diseases; For female patients, 962 cases (54.1%) were tumor diseases and 89 cases (5.0%) were infectious diseases (Table 1).

### Anesthesia

**Fig. 3:**
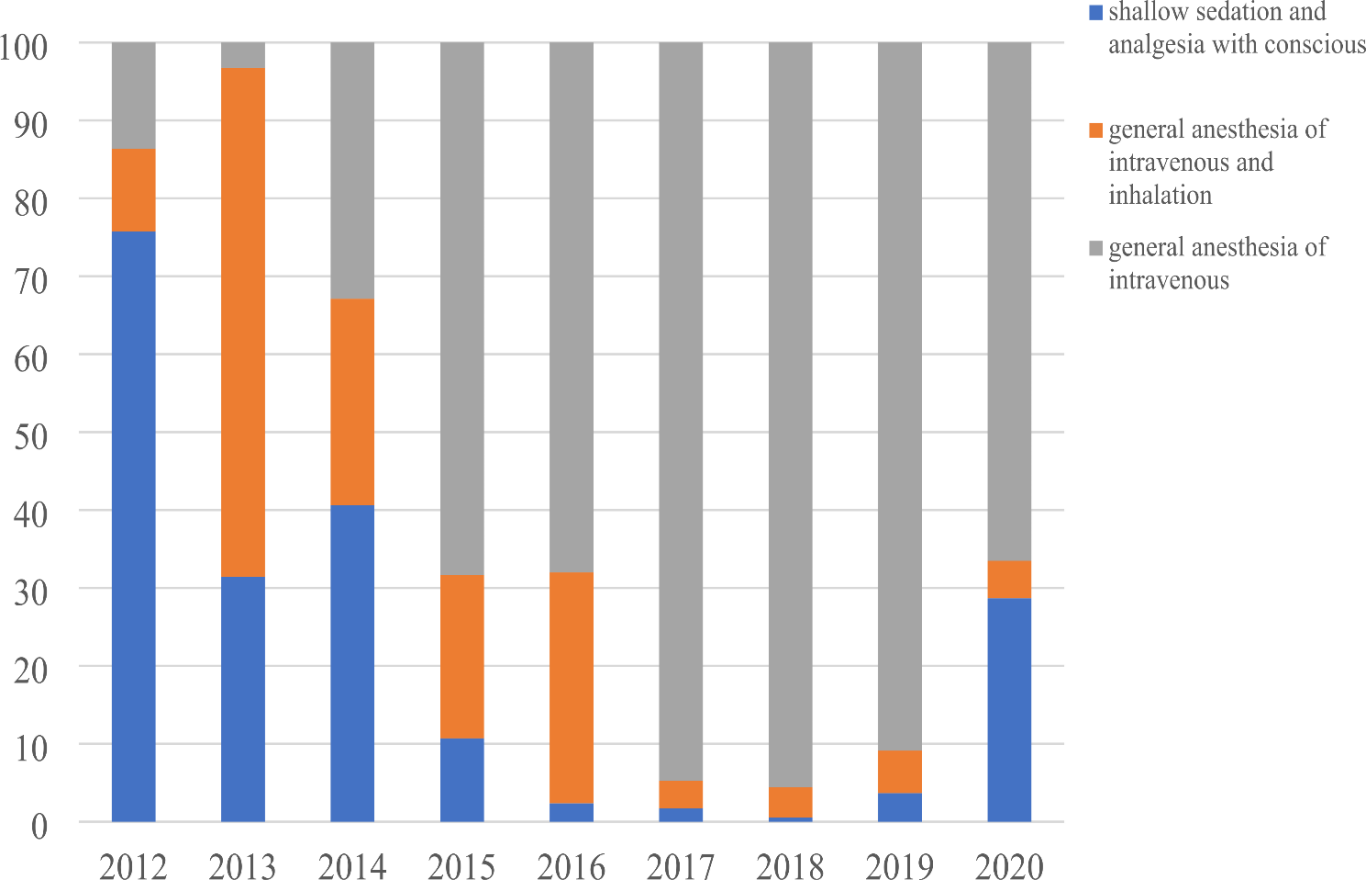
Anesthesia methods used in different years

All patients were anesthetized during the tests, included deep general anesthesia of intravenous and inhalation with tracheal intubation and mechanical ventilation in 996 cases (17.9%), general anesthesia of intravenous with spontaneous breathing in 3867 cases (69.4%), and shallow sedation and analgesia with conscious spontaneous breathing in 713 cases (12.8%). Follow the years, more and more patients anesthetized by general intravenous anesthesia with spontaneous breathing (more than 90% since 2017), and less patients anesthetized by deep general anesthesia of intravenous and inhalation with tracheal intubation and mechanical ventilation (Fig. 3). General intravenous anesthesia with spontaneous breathing accounted for the highest proportion of anesthesia methods in different kind of diseases (Table 3).

**Table 3:** Anesthesia methods used in different disease of patients

Anesthesia /Disease	Tumor N(%)	Infectious N(%)	Sarcoidosis N(%)	Lymphoma N(%)	Others N(%)	p
general anesthesia of intravenous and inhalation	666(18.0)	115(20.8)	47(21.9)	10(16.4)	158(15.2)	< 0.01
general anesthesia of intravenous	2544(68.7)	371(67.0)	143(66.5)	39(63.9)	770(74.0)	< 0.01
shallow sedation and analgesia with conscious	495(13.4)	68(12.3)	25(11.6)	12(19.7)	113(10.8)	< 0.01
Total	3705	554	215	61	1041	

### EBUS-TBNA diagnostic value

**Fig. 4:**
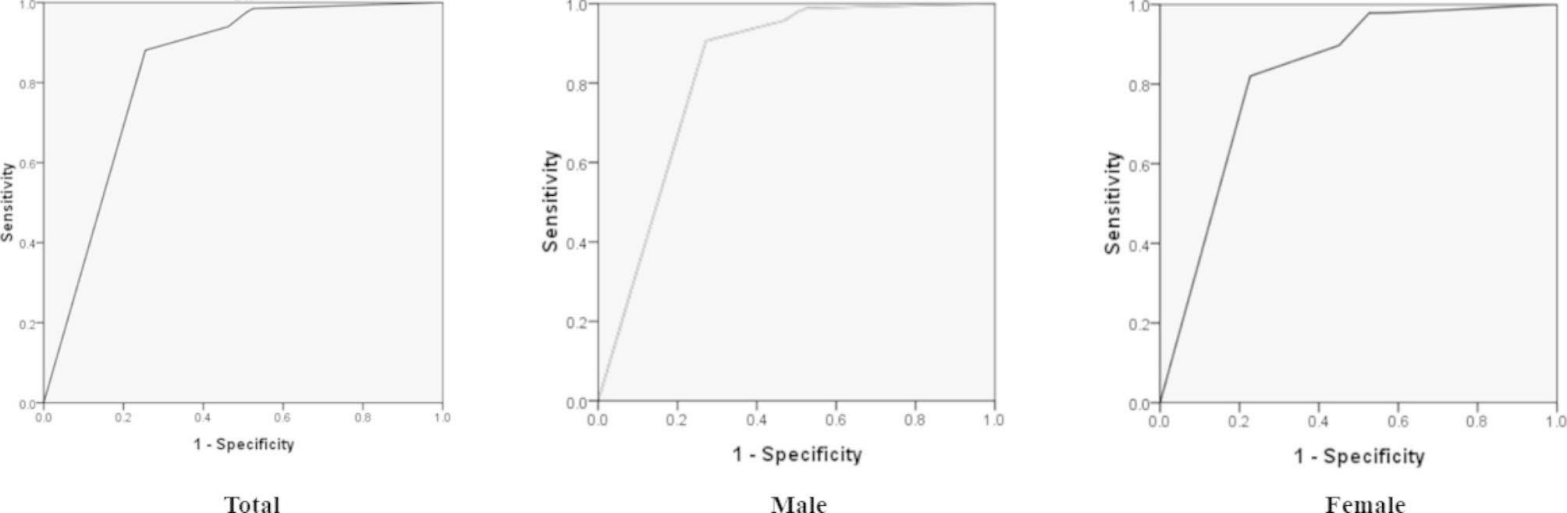
ROC curve AUC: total: 0.833, male: 0.834, female: 0.824

The overall diagnostic sensitivity was 67.9% for all patients, and the sensitivity was 70.2% for male patients, 62.9% for female patients. The area under the curve (AUC) was 0.915 for male patients, and 0.890 for female patients (Fig. 4). The positive rate of pathological diagnosis by EBUS-TBNA was 89.3% (3307 cases) in tumor patients, and 37.4% (207 cases) in patients of pulmonary infectious diseases. Of the infection diseases, 77.6% were mycobacterial infection, 8.4% fungal infection, and 14.0% of other pulmonary infections.

### Complications

There had no severe adverse effects reported in this study, and some of the patients reported with mild to moderate symptoms, like cough with or without bloody sputum, which recovered after hours to few days.

## Discussion

In this retrospective study, we analyzed the data of 5576 patients who had completed EBUS-TBNA in the center during the past 10 years. The results showed that the number of patients underwent EBUS-TBNA increased over years of time, demonstrated the increased need of EBUS-TBNA in clinical. As recommended [1, 2], the patients with central lung cancer are the most important indications for EBUS-TBNA, Recently, a survey in India reported, suspected granulomatous mediastinal lymphadenopathy (TB/sarcoidosis) (67.2%) and lung cancer (32.8%) were the most common indications of EBUS-TBNA, which were different from our data [13]. Moreover, in this study, it showed that, besides the highest proportion of tumor patients, it was also applied for the diagnosis of different kind of diseases, like, infection diseases, sarcoidosis and lymphoma, et al. As we know, EBUS-TBNA is guided by an ultrasound bronchoscope to obtain real-time structural imaging of the bronchial wall and extramural tissue to clarify the location and character of the lesion, and at the same time, it can display the blood supply of the target or surrounding the lesion to avoid bleeding caused by puncturing the blood vessel by mistake. Under the guidance, a disposable suction biopsy needle is used for puncture or suction to obtain a biopsy sample. Because of its minimally invasive characteristics, good preoperative anesthesia intervention, and good tolerance by patients, this technology can be promoted in clinical practice [13]. So, EBUS-TBNA can be used for the diagnosis of different kind of diseases that the lesions can be reached [14, 15].

The overall diagnostic sensitivity was 67.9% of all patients, with highest positive rate (89.3%) in lung cancer patients, higher than previous reports [4, 11]. The positive rate of pulmonary infectious diseases was 37.4%, however, there had not include the results of bronchoalveolar lavage fluid (BALF) for etiology, which may highly increase the diagnostic sensitive rate. Mycobacterial infection accounted for the highest proportion of the infection diseases; Most of these patients have unexplained mediastinal lymphadenopathy, which is easily confused with tumor and needs to be clearly identified. Recent literature also reported that EBUS-TBNA has a good diagnostic value for mediastinal tuberculosis [15–17]. As recommended in the guideline, EBUS-TBNA has been used for initial evaluation (diagnosis, staging, identification of recurrence/metastasis) of mediastinal and hilar lymph nodes, as well as centrally located parenchymal lesions visible with endobronchial ultrasound. For some cases, the sample was difficult to get, and for some kind of disease, the results of pathology was used for differential diagnosis, or it was not the gold standard for the diagnostic. So the most suitable candidates for EBUS-TBNA were tumor in mediastinal and hilar lymph nodes, as well as centrally located parenchymal lesions.

In this study, higher diagnostic yield was found in male patients. The data showed that there was a significant difference of the proportion of male and female patients, there were much more male than female patients, which might caused by the different kind of diseases. There was higher morbidity of lung cancer of male than female, while the proportion of patients with infectious diseases higher in females. For the higher sensitivity of TBNA in the diagnosis of lung cancer, totally the diagnosis sensitivity was 70.2% of male patients, and 62.9% of female patients. Previous studies also showed that there were little higher percent of males than females that underwent TBNA [18–20]. There were few studies compared the diagnostic value of EBUS-TBNA between male and female patients.

In this study, lymph nodes were the main sites obtained, with highest percent in sarcoidosis patients, however, the positive rate was slightly higher obtained in lung lesions than that of lymph nodes. Some studies have discussed the application of EBUS-TBNA in the diagnosis of mediastinal enlarged lymph nodes and central pulmonary lesions [4, 11]. In this study, a little proportion of patients had both lymph nodes and intrapulmonary lesions punctured with positive rate (60.4%) lower than those patients with lymph node and intrapulmonary lesion obtained only, which might be for the difficulty of target or lesion been punctured of these patients. EBUS-TBNA can be used in different kind of diseases that the lesions can be reached [14, 15]. However, the choose of puncture site might depend of the disease and location of the lesion.

Anesthesia is very important to protect the patients to tolerate the operation of EBUS-TBNA [13, 19, 21]. In this study, the anesthesia method was mainly general intravenous anesthesia with spontaneous breathing, which was safe with little trauma of the patient and caused less adverse effects. Most of the patients can tolerate the operation well, even in elder patients that more than 80 years old [20, 22, 23]. The operation can also be completed by mild anesthesia of sedation and analgesia with the patients awake, which is suitable for some patients with poor cardiopulmonary function or who cannot tolerate deep anesthesia [19]. There was an interesting trends in this study that more and more patients anesthetized by general intravenous anesthesia with spontaneous breathing (more than 90% since 2017), and less patients anesthetized by deep general anesthesia of intravenous and inhalation with tracheal intubation and mechanical ventilation follow the years, which consisted with the results reported in previous study that EBUS-TBNA performed under conscious sedation with meperidine and midazolam is feasible and well-tolerated and has a good diagnostic yield [19].

There were few limitations of this study, first, in this retrospective study, clinical application of EBUS-TBNA in the diagnosis of different diseases were described, we need some prospectively comparisons of the diagnostic yield of EBUS-TBNA in different diseases; Second, the different positive rate between male and female patients may be caused by different proportions of patients with different diseases, and case-control study or paired comparisons should be performed; Third, more concern should be paid on the complications of EBUS-TBNA.

In conclusion, EBUS-TBNA has been mainly used for the diagnosis of mediastinal and hilar lesions diseases, however, other diseases were also good candidates, like, infectious diseases, sarcoidosis and lymphoma. Under mild anesthesia, most patient can tolerant EBUS-TBNA, and the punctured site can be enlarged lymph nodes and pulmonary lesions. There was a difference in the diagnostic sensitivity of EBUS-TBNA between male and female patients, which need more study and subgroup analysis.

## Data Availability

Data of this study can be got by anyone who contact to the author Chun-li Tang (tcl8182@163.com) by E-mail for studying use.

## References

[1] Griffin JP, Koch KA, Nelson JE, Cooley ME. American College of Chest Physicians. Palliative care consultation, quality-of-life measurements, and bereavement for end-of-life care in patients with lung cancer: ACCP evidence-based clinical practice quidelines (2nd edition). Chest. 2007;132(3 Suppl):404S-422S. 10.1378/chest.07-1392 17873182

[2] Kvale PA, Selecky PA, Prakash UB. ; American College of Chest Physicians.Palliative care in lung cancer: ACCP evidence-based clinical practice guidelines (2nd edition). Chest. 2007;132(3 Suppl):368S-403S. 10.1378/chest.07-1391 17873181

[3] Wang Guangfa C, Maosen L. Guilian, Findings and diagnostic value of fiberoptic bronchoscopy in sarcoidosis. Chin J endoscopy, 1999, (4): 16–7.

[4] Zhang H, Guangfa W, Wei Z (2014). Diagnostic value of ultrasound-guided transbronchial needle aspiration biopsy in sarcoidosis. Chin J tuberculosis respiration.

[5] Reynolds HY (2009). Present status of bronchoalveolar lavage in interstitial lung disease. Curr Opin Pulm Med.

[6] Leonard C, Tormey VJ, O′Keane C (1997). Bronchoscopic diagnosis of sarcoidosis. Eur Respir J.

[7] Puar HS, Young RC, Armstrong EM (1985). Bronchial and transbronchial lung biopsy without fluoroscopy in sarcoidosis. Chest.

[8] Shorr AF, Torrington KG, Hnatiuk OW (2001). Endobronchial biopsy for sarcoidosis: a prospective study. Chest.

[9] Rohatgi PK, Kuzmowych TV, Delaney MD (1981). Indications for transbronchial lung biopsy in the diagnosis of intrathoracic sarcoidosis. Respiration.

[10] Navani N, Booth HL, Kocjan G (2011). Combination of endobronchial ultrasound-guided transbronchial needle aspiration with standard bronchoscopic techniques for the diagnosis of stage I and stage II pulmonary sarcoidosis. Respirology.

[11] Xie Zhen Z, Hui Z, Zuli (2013). Application value of endobronchial ultrasound-guided needle aspiration biopsy in the diagnosis and differential diagnosis of mediastinal lesions. Chin J Minim invasive Surg.

[12] Kang N, Shin SH, Yoo H, Jhun BW, Lee K, Um SW, Kim H, Jeong BH (2021). Infectious complications of EBUS-TBNA: a nested case-control study using 10-year registry data. Lung Cancer.

[13] Muthu V, Sehgal IS, Dhooria S, Prasad KT, Gupta N, Aggarwal AN, Agarwal R (2019). Endobronchial ultrasound-guided transbronchial needle aspiration: Techniques and Challenges. J Cytol.

[14] Rosso L, Ferrero S, Mendogni P, Bonaparte E, Carrinola R, Palleschi A, Righi I, Montoli M, Damarco F, Tosi D (2017). Ten-year experience with endobronchial ultrasound-guided transbronchial needle aspiration: single center results in mediastinal diagnostic and staging. J Thorac Dis.

[15] Jeebun V, Harrison RN (2017). Understanding local performance data for EBUS-TBNA: insights from an unselected case series at a high-volume UK center. J Thorac Dis.

[16] Lucey O, Potter J, Ricketts W, Castle L, Melzer M (2022). Utility of EBUS-TBNA in diagnosing mediastinal tuberculous lymphadenitis in East London. J Infect.

[17] Cheng LP, Gui XW, Fang Y, Sha W, Gu Y (2021). Clinical value of endobronchial ultrasound-guided aspiration and local isoniazid injection in the treatment of mediastinal tuberculous lymphadenitis. Ann Palliat Med.

[18] Özbudak Ö, Dirol H, Öngüç İ, Kahraman H (2021). Is ASA classification useful in risk stratification for EBUS-TBNA?. Turk Thorac J.

[19] Piro R, Casalini E, Fontana M, Galeone C, Ruggiero P, Taddei S, Ghidoni G, Patricelli G, Facciolongo N (2022). Efficacy and safety of EBUS-TBNA under conscious sedation with meperidine and midazolam. Thorac Cancer.

[20] Levin VG, Romem A, Epstein Shochet G, Wand O, Dahan D, Shitrit D (2022). The Diagnostic yield of Endobronchial Ultrasound Transbronchial needle aspiration (EBUS-TBNA) in respiratory compromised patients under General Anesthesia. Isr Med Assoc J.

[21] Madan K, Mittal S, Tiwari P, Hadda V, Mohan A, Guleria R (2022). A survey of Endobronchial Ultrasound-guided Transbronchial needle aspiration (EBUS-TBNA practices in India. Lung India.

[22] Niwa H, Oki M, Ishii Y, Torii A, Yamada A, Shinohara Y, Kogure Y, Saka H (2022). Safety and efficacy of endobronchial ultrasound-guided transbronchial needle aspiration (EBUS-TBNA) for patients aged 80 years and older. Thorac Cancer.

[23] Bergbower EAS, Hong C, Gibbons M, Sachdeva A, Rock P, Anders MG (2022). A retrospective analysis of respiratory complications under General Anesthesia during EBUS-TBNA. J Community Hosp Intern Med Perspect.

